# Graphene oxide-modified silk fibroin/nanohydroxyapatite scaffold loaded with urine-derived stem cells for immunomodulation and bone regeneration

**DOI:** 10.1186/s13287-021-02634-w

**Published:** 2021-12-04

**Authors:** Jiachen Sun, Lang Li, Fei Xing, Yun Yang, Min Gong, Guoming Liu, Shuang Wu, Rong Luo, Xin Duan, Ming Liu, Min Zou, Zhou Xiang

**Affiliations:** 1 Department of Orthopedics, West China Hospital, Sichuan University, Guoxue Lane 37, Chengdu, 610041 Sichuan Province People’s Republic of China; 2 Department of Orthopedics, Hospital of Chengdu Office of People’s Government of Tibetan Autonomous Region, Chengdu, 610041 Sichuan People’s Republic of China; 3 Department of Orthopedics, Hospital of Chengdu University of Traditional Chinese Medicine, Chengdu, 610075 Sichuan People’s Republic of China; 4 Department of Orthopedics, Affiliated Hospital of Qingdao University, Qingdao, 266003 Shangdong People’s Republic of China; 5 Department of Orthopedics, Chengdu Second People’s Hospital, Chengdu, 610017 Sichuan People’s Republic of China

**Keywords:** Urine-derived stem cells, Graphene oxide, Macrophages, Immunomodulation, Bone repair

## Abstract

**Background:**

The invasive and complicated procedures involving the use of traditional stem cells limit their application in bone tissue engineering. Cell-free, tissue-engineered bones often have complex scaffold structures and are usually engineered using several growth factors (GFs), thus leading to costly and difficult preparations. Urine-derived stem cells (USCs), a type of autologous stem cell isolated noninvasively and with minimum cost, are expected to solve the typical problems of using traditional stem cells to engineer bones. In this study, a graphene oxide (GO)-modified silk fibroin (SF)/nanohydroxyapatite (nHA) scaffold loaded with USCs was developed for immunomodulation and bone regeneration.

**Methods:**

The SF/nHA scaffolds were prepared via lyophilization and cross-linked with GO using 1-ethyl-3-(3-dimethylaminopropyl) carbodiimide hydrochloride (EDC) and N-hydroxy succinimide (NHS). Scaffolds containing various concentrations of GO were characterized using scanning electron microscopy (SEM), the elastic modulus test, Fourier transform infrared spectroscopy (FTIR), and X-ray photoelectron spectrometer (XPS). Examinations of cell adhesion, proliferation, viability, morphology, alkaline phosphatase activity, and osteogenesis-related gene expression were performed to compare the osteogenesis-related biological behaviors of USCs cultured on the scaffolds. The effect of USC-laden scaffolds on the differentiation of macrophages was tested using ELISA, qRT-PCR, and immunofluorescence staining. Subcutaneous implantations in rats were performed to evaluate the inflammatory response of the USC-laden scaffolds after implantation. The scaffolds loaded with USCs were implanted into a cranial defect model in rats to repair bone defects. Micro-computed tomography (μCT) analyses and histological evaluation were performed to evaluate the bone repair effects.

**Results:**

GO modification enhanced the mechanical properties of the scaffolds. Scaffolds containing less than 0.5% GO had good biocompatibility and promoted USC proliferation and osteogenesis. The scaffolds loaded with USCs induced the M2-type differentiation and inhibited the M1-type differentiation of macrophages. The USC-laden scaffolds containing 0.1% GO exhibited the best capacity for promoting the M2-type differentiation of macrophages and accelerating bone regeneration and almost bridged the site of the rat cranial defects at 12 weeks after surgery.

**Conclusions:**

This composite system has the capacity for immunomodulation and the promotion of bone regeneration and shows promising potential for clinical applications of USC-based, tissue-engineered bones.

**Supplementary Information:**

The online version contains supplementary material available at 10.1186/s13287-021-02634-w.

## Background

Reconstructing bone defects remains a challenge in the clinical setting [1, 2]. As the gold standard therapy, transplantation with autologous bone grafts is limited by low harvest and availability and donor site pain. The risk of infection and rejection also limits the application of allografts [3, 4]. Tissue-engineered bone has the promise to become an alternative strategy to solve these problems [5, 6]. The key elements of traditional tissue engineering include seeding cells, scaffolds, and growth factors (GFs). The traditional seeding cells used in bone tissue engineering include stem cells (SCs) from various sources, such as bone marrow mesenchymal stem cells (BMSCs), adipose-derived stem cells (ADSCs), and induced pluripotent stem cells (iPSCs) [7–9]. Recently, however, many studies on tissue-engineered bone have abandoned the use of seeding cells because of scarce sources, the invasive procedures used to obtain them, and the occurrence of tumorigenesis after implantation [10, 11]. These cell-free, tissue-engineered bones often have complex scaffold designs simulating the structure of natural bone and are usually incorporated with several GFs to promote cell homing, proliferation, and osteogenic differentiation of the repair-related cells [12, 13]. As a result, the production of cell-free, tissue-engineered bones is costly and difficult, thus rendering their use in clinical applications imprudent.

Recently, some studies have shown that the promotion of tissue repair via SCs’ transplantation is also related to the regulation of local immune responses [14–16]. Macrophages are one of the most important effector cells involved with the biomaterial-induced immune response [17]. They can be generally divided into the proinflammatory M1 phenotype and the antiinflammatory M2 phenotype. M1-type macrophages can secrete proinflammatory cytokines, such as tumor necrosis factor-α (TNF-α) and interleukin-6 (IL-6), to promote an inflammatory response, while M2-type macrophages can secrete antiinflammatory cytokines, such as interleukin-10 (IL-10) and transforming growth factor-β (TGF-β), to inhibit inflammation and promote tissue repair [18]. SCs’ implantation can reduce local inflammation and accelerate tissue regeneration by promoting the differentiation of macrophages into the M2 phenotype [14, 16]. Therefore, the application of SCs still plays an important role in tissue engineering. Unlike traditional SCs, urine-derived stem cells (USCs) are expected to solve the challenges of seeding cells and improve clinical applications of tissue-engineered bones. USCs can be isolated from human urine with little ethical controversy, and they possess SC-like properties [19]. They can be repeatedly and noninvasively obtained from the same individual and used for autologous implantation to reduce immune rejection [20]. To date, USCs have been used in tissue engineering involving the repair of urethras, kidneys, myocardia, cartilage, and bone [21–25]. In these studies, no obvious immune rejection or tumorigenesis was observed, which preliminarily proved the low immunogenicity and the xenotransplantation safety of USCs. Although some studies reported that USCs showed lower osteogenic capacity compared with BMSCs or ADSCs, this shortcoming can be overcome by optimizing the composition and design of the scaffold [26, 27].

Graphene oxide (GO) is a member of graphene family materials, materials that are among the thinnest in the world, and it is widely used in tissue engineering [28, 29]. It has a large specific surface area, numerous functional groups, and excellent hydrophilicity, all of which make it conducive to bonding covalently, noncovalently, and electrostatically to scaffold materials [30, 31]. GO modification is usually used to improve the biomechanical properties of scaffolds; promote cell adhesion, proliferation, and differentiation; and act as a drug delivery system [32, 33]. Hence, GO modification has the potential to enhance the osteogenesis-related capacities of USCs. Additionally, hydroxyapatite is the main component of natural bone and has osteoinductive ability [34]. Nano-hydroxyapatite (nHA) is widely used in tissue-engineered bone, often to improve osteogenic capacity [35], and is inexpensive. Wang et al. cross-linked GO with hydroxyapatite (HA) to enhance the osteogenic capacity of HA particles [36]. They adjusted the GO–HA concentration in the silk fibroin (SF) scaffold to obtain a graded scaffold to imitate the dense, cancellous, and sponge layers of the natural bone. The obtained scaffold presented improved biomechanical properties and osteogenic capacity compared with scaffolds loaded with only HA. Moreover, SF is a natural polymer product approved by the Food and Drug Administration for clinical medicine and displays adjustable mechanical properties and biodegradation and good preservation of biomolecule activity [37]. It is inexpensive and easy to obtain and can be chemically modified. It has been demonstrated to be a favorable scaffold material for tissue-engineered bone, and SF scaffolds incorporated with nHA supported cell growth and osteogenic differentiation of BMSCs [38, 39]. Therefore, the combination of GO, nHA, and SF is promising for the construction of a scaffold to load with USCs for bone repair.

In this study, we developed a novel scaffold for local immunomodulation and bone regeneration. We incorporated nHA into SF scaffolds and modified the scaffolds with GO. Various concentrations of GO were tested to determine an optimal GO concentration to optimize the scaffold’s physical properties and enhance the loaded USCs’ osteogenesis-related capacities. We evaluated the effects of the USC-laden GO–SF/nHA scaffolds on immunomodulation and bone repair using a rat subcutaneous implantation model and a rat calvarial defect model, respectively.

## Methods

The whole experimental protocol was approved by the Animal Care and Experiment Committee of West China Hospital affiliated to Sichuan University, Chengdu, China (2020228A). All the reagents were purchased from SigmaAldrich (USA), the antibodies were purchased from Abcam (UK), the cell culture medium was supplied by Gibco (Billings, MT, USA), and the cell induction medium was supplied by Cyagen (Guangzhou, China) unless otherwise stated.

### Preparation of GO-modified SF/nHA scaffolds

The SF solution was prepared from cocoons of *B. mori* (RudongXinsilu Co., Ltd., Jiangsu, China) using the method outlined in our previous report [40]. The GO powder (~ 50 μm, single-layer; Timesnano, Chengdu, China) was used to prepare a solution with ultrapure water via ultrasonic dispersion for 2 h. The GO sheets after dispersion were detected using a transmission electron microscope (TEM; Tecnai G2 F20 S-TWIN, FEI, USA). The GO solution and SF solution were mixed and stirred for 4 h at room temperature. The nHA powder (50–100 nm; Aladdin, Shanghai, China) was used to prepare a suspension with PBS via ultrasonic dispersion for 5 min. Carboxylated cellulose nanofibrils (cCNF) (HaoJia Nanofibrils technology Co., Ltd, Tianjing, China) were used in the nHA suspension at a ratio of 4:1 (*v*/*v*) to improve the stability of nHA. The nHA/cCNF suspension was mixed with the GO/SF solution and added into a 96-well plate (280 μL/well). The ultimate concentration of SF was 6% (*w*/*v*), the mass ratio of SF/nHA was 10:1, and the ultimate concentration of GO (*w*/*w*) was 0%, 0.01%, 0.05%, 0.1%, 0.5%, and 1%. The plate was frozen in increments, first for 12 h at − 20 °C and then for 12 h at − 80 °C, and finally the plate was freeze-dried for 24 h. Next, the preliminary scaffolds were treated with methanol for 30 min to induce β-sheet transformation of SF. Finally, the scaffolds were cross-linked using 50 mM 1-ethyl-3-(3-dimethylaminopropyl) carbodiimide hydrochloride (EDC) and 20 mM N-hydroxy succinimide (NHS) (pH 4.5–5.0) for 4 h at room temperature and then rinsed with ultrapure water to remove any excess reagent. After lyophilization for 24 h, the final scaffolds containing different concentrations of GO were labeled “0%,” “0.01%,” “0.05%,” “0.1%,” “0.5%,” and “1%.” The uncross-linked scaffolds without GO were labeled “uncross-linked” and used to compare the characterization differences with the cross-linked scaffolds. All the scaffolds were cut into cylindrical slices 5 mm in diameter and 1.5 mm in thickness prior to use unless otherwise stated. The scaffolds were sterilized using ethylene oxide before the in vitro and in vivo experiments.

### Characterization of scaffolds

#### Microstructure analyses

The morphology of the scaffolds was observed using scanning electron microscopy (SEM) (S-4800; Hitachi, Kyoto, Japan) at 20 kV. The samples were sputtered with gold for 60 s using gold sputter coating equipment (SC7620, Quorum Technologies, UK). For each sample, we randomly selected three visual fields, and the pore size of each scaffold sample was measured using Image J software. The average porosity of the scaffolds was measured via liquid displacement using hexane.

#### Evaluation of elastic modulus and degradation

Unsliced scaffolds were used for both the elastic modulus and degradation tests. The compressive modulus of elasticity was measured using 500 N force at a loading velocity of 1 mm/min (Instron, Norwood, MA, USA). The initial weights of the scaffolds (M0) were recorded, followed by incubation in simulated body fluid (SBF) at 37 °C in a shaking table at 100 r min^−1^. The SBF was replaced every 3 days. At defined time points (7, 14, 28, and 56 days), the scaffolds were removed from the SBF, dried with absorbent paper, lyophilized, and reweighed (*M*1). Degradation was quantified as the change in the sample’s weight over time. The remaining weights of the scaffolds were calculated as follows: degradation rate (%) = (*M*0 − *M*1)/*M*0 × 100%.

#### Analysis of FTIR and XPS

The main functional groups of the scaffolds were investigated by Fourier transform infrared spectroscopy (FTIR) (Nicolet 6700; Thermo Scientific, USA). The scaffolds were ground into powder with potassium bromide, pressed into tablets, and tested using transmission method. FTIR measurements were conducted at a resolution of 4 cm^−1^ from 400 to 4000 cm^−1^. The surface elements of the scaffolds were determined using an X-ray photoelectron spectrometer (XPS) (AXIS Supra; Kratos, UK). For each sample, a narrow scan of carbon elements was prepared (O, 524–544 eV; C, 280–300 eV; N, 392–412 eV), and the measurement was conducted at a takeoff angle of 90°.

### Effect of scaffolds on the osteogenesis-related biological behaviors of USCs

#### Culture of USCs

The USCs were identified and provided by Dr. Fei Xing [41]. The third, fourth, and fifth passage USCs were used in this study. The culture medium contained 50% keratinocyte serum-free medium (K-SFM), 32.75% Dulbecco’s modified Eagle’s medium–high glucose (DMEM-HG), 11.25% Ham’s F12 (HyClone, Logan, UT, USA), 5% fetal bovine serum (FBS), 1% penicillin–streptomycin solution, and several supplements (5 ng/mL epidermal growth factor [EGF], 50 ng/mL bovine pituitary extract [BPE], 0.4 μg/mL hydrocortisone, 5 μg/mL transferrin, 5 ng/mL bovine insulin, 0.18 mmol/L adenine, and 2 nmol/L 3,3,5-triiodo-L-thyromine). The cells were incubated in 5% CO_2_ at 95% humidity and 37 °C. The medium was replaced every 2 days.

#### Assay of cytotoxicity and hemolysis

A cytotoxicity assay was performed using the extraction media of the scaffolds. A 10 mL volume of culture medium containing 1 g of scaffold was incubated in 5% CO_2_ for 48 h at 37 °C. The extraction medium was collected by centrifugation at 4000×*g* for 15 min, filtered through a 0.75 μm sterile mesh filter, and stored at − 20 °C. USCs were suspended in 20 μL of culture medium and seeded onto a 96-well plate at a density of 5 × 10^3^, and then 200 μL of the extraction medium was added. The medium was changed every 2 days. Cell Counting Kit-8 (CCK-8) tests (Dojindo, Japan) were performed according to the instructions after culturing for 1, 3, and 5 days.

Fresh ethylenediaminetetraacetic acid (EDTA)-stabilized rabbit whole blood samples were provided by Dr. Fei Xing when he needed to sacrifice the New Zealand white rabbits for another animal experiment. A 5 mL volume of whole blood was added to 10 mL of PBS and centrifuged at 500×*g* for 10 min to isolate the red blood cells (RBCs). This purification step was repeated 3 times, and then the washed RBCs were diluted to 50 mL in PBS. The scaffold was added into a 1.5-mL Eppendorf tube with 200 μL of the diluted RBC suspension. Water and PBS were added to the RBC suspension as the positive control (+) and negative control (−), respectively. The samples were placed on a rocking shaker in an incubator at 37 °C for 3 h. After incubation, the samples were centrifuged at 10,016×*g* for 3 min. The hemoglobin absorbance in the supernatant was measured at 545 nm.

#### Test of cell adhesion, proliferation, viability, and morphology

The scaffolds were placed in 48-well plates and soaked in DMEM-HG for 24 h. The DMEM-HG was removed, and 1 mL of culture medium containing 1 × 10^4^ USCs was seeded onto each scaffold. After 1, 2, and 4 h of culture, the scaffolds were washed with PBS, and the adhered cells were quantified using the CyQuant assay kit (Thermo Fisher Scientific) based on DNA fluorescence.

A volume of 200 μL of culture medium containing 5 × 10^3^ USCs was seeded onto each scaffold placed in a 96-well plate. The medium was replaced every 2 days. At desired time points (1, 4, and 7 days), the cell proliferation of each sample was evaluated using the CyQuant assay kit. After 7 days of culture, the cell viability was tested using a live–dead staining kit (Yisheng, Shanghai, China) according to the instructions. At the same time, SEM examination was performed to observe cell morphology. The samples were dehydrated using a gradient series of ethanol/water solutions (10%, 20%, 35%, 50%, 70%, 85%, and 100%) and dried using the CO_2_ critical-point drying method. The SEM was operated at 20 kV to image the samples.

#### Examination of in vitro osteogenesis

USCs numbering 1.5 × 10^5^ were seeded onto each scaffold placed in a 48-well plate and cultured with culture medium for 3 days. Then, the culture medium was replaced with osteogenic induction medium. The induction medium was changed every 3 days. After 10 days of induction, cells were lysed with 0.1% Triton X-100 and tested using an alkaline phosphatase activity (ALP) kit (Jiancheng, Nanjing, China) according to the instructions. After 7 and 21 days of induction, quantitative reverse transcription polymerase chain reaction (qRT-PCR) was performed to analyze the osteogenesis-related gene expression of the samples, as previously described [42]. In order to provide sufficient cells for PCR detection, each group included three samples, and each sample was composed of three scaffolds. Osteogenesis-related genes included *ALP*, runt-related factor-2 (*Runx2*), osteocalcin (*OCN*), and osteopontin (*Opn*). Glyceraldehyde 3-phosphate dehydrogenase (*GAPDH*) was used as an internal control. All primers were synthesized by Sangon Biotech (Shanghai, China) (Table 1).

**Table 1:** Primer sequences used for qRT-PCR

Gene	Primer/probe	Sequence
*ALP*	Forward	ACCACCACGAGAGTGAACCA
	Reverse	CGTTGTCTGAGTACCAGTCCC
*Runx2*	Forward	CCAACCCACGAATGCACTATC
	Reverse	TAGTGAGTGGTGGCGGACATAC
*OCN*	Forward	CCCCCTCTAGCCTAGGACC
	Reverse	ACCAGGTAATGCCAGTTTGC
*OPN*	Forward	GACGAGCACATCACCTCACA
	Reverse	GGCTTCAGCACTCTGGTCAT
*GAPDH*	Forward	ACAACTTTGGTATCGTGGAAGG
	Reverse	GCCATCACGCCACAGTTTC

### Effect of scaffolds loaded with USCs on the differentiation of macrophages

To evaluate the effect of the scaffolds on the differentiation of macrophages, the scaffold containing 0.1% GO, which significantly promoted USC proliferation and osteogenic differentiation, was selected for follow-up experiments. The scaffolds without GO were labeled “control,” the GO-free scaffolds loaded with 1.5 × 10^5^ USCs were labeled “U,” the scaffolds containing 0.1% GO were labeled “GO,” and the scaffolds containing 0.1% GO loaded with 1.5 × 10^5^ USCs were labeled “GO+U.” Rat peritoneal macrophages were used in this study because of the low immunogenicity of USCs. The macrophages were identified and provided by Dr. Min Zou [43]. Macrophages numbering 2 × 10^5^ were seeded in the lower chamber of a 24-well Transwell plate (0.4 μm, Corning, USA) in macrophage culture medium. After 2 h of incubation, unattached cells were removed, and the scaffold was placed in the upper chamber for coculture. IL-4/IL-10/TGF-β were applied at final concentrations of 20 ng mL^−1^ to treat macrophages as a positive control (+), and macrophages cultured with only culture medium were labeled “Blank.” After 48 h of coculture, the supernatant was collected and centrifuged to detect the protein expression of TNF-α and IL-10 using ELISA kits (R&D Systems, USA). At the same time, qRT-PCR was performed to detect the gene expression of the macrophages, including inducible nitric oxide synthase (*iNOS*) and *TNF-α* for M1-type macrophages and *CD206* and arginase-1 (*Arg-1*) for M2-type macrophages. *GAPDH* was used as an internal control. The primers were synthesized by Sangon Biotech (Table 2).

**Table 2:** Primer sequences used for qRT-PCR

Gene	Primer/probe	Sequence
*iNOS*	Forward	ATTCACTCAGCTGTGCATCG
	Reverse	TCAGGTGGGATTTCGAAGAG
*TNF-α*	Forward	GCTCTTCTGTCTACTGAACTTCGG
	Reverse	ATGATCTGAGTGTGAGGGTCTGG
*CD206*	Forward	GCTTGTAGGAAGGAGGGT
	Reverse	TCCAGGAAGCCATTTAGT
*Arg-1*	Forward	CTCCAAGCCAAAGTCCTTAGAG
	Reverse	AGGAGCTGTCATTAGGGACATC
*GAPDH*	Forward	CTGCACCACCAACTGCTTAG
	Reverse	GTCTGGGATGGAAATTGTGA

CD206 immunofluorescence staining was also performed to verify the M2-type differentiation of macrophages. The macrophages were seeded onto coverslips and then placed in the lower chamber of the 24-well Transwell plate. After 48 h of coculture, the coverslips were collected, fixed with 4% paraformaldehyde, treated with 0.5% Triton X-100, and blocked with 1% bovine serum albumin. After washing with PBS, the cells were stained against the CD206 primary antibody overnight and then incubated in the secondary antibody. The nuclei were stained with 4′,6-diamidino-2-phenylindole (DAPI).

### Subcutaneous implantation in rats

To evaluate the inflammatory response of the scaffolds after implantation, tests of the subcutaneous implantations in rats were performed. Eighteen 12-week-old male Sprague–Dawley (SD) rats were randomly assigned to four groups. The rats were anesthetized using chloral hydrate, and a bilateral subcutaneous implantation model was established on the back of the rats. Longitudinal skin incisions 1 cm in length were made, and the scaffolds were implanted into the model. The scaffolds without GO were labeled “control,” the GO-free scaffolds loaded with 5 × 10^5^ USCs were labeled “U,” the scaffolds containing 0.1% GO were labeled “GO,” and the scaffolds containing 0.1% GO loaded with 5 × 10^5^ USCs were labeled “GO+U.” The rats were sacrificed by an overdose of anesthesia after 3, 7, and 14 days of implantation, and specimens were collected, fixed, and stained with hematoxylin and eosin (H&E), CD68 (a specific marker of macrophages), iNOS, and CD206.

### Bone repair of calvarium in rats

Twenty 12-week-old male SD rats were randomly assigned to five groups and a bilateral cranial defect model was established. After anesthesia using chloral hydrate, a 2.0 cm incision in the sagittal direction was made, the periosteum was removed, and 5 mm diameter defects were created using a dental trephine drill. Then, the four aforementioned groups of scaffolds were implanted into the model, and the rats without a scaffold implantation were labeled “Blank.” Finally, the incision was closed with 4-0 sutures. The rats were sacrificed by an overdose of anesthesia at 6 and 12 weeks after surgery. Cranial specimens were collected for micro-CT (Quantum GX, PerkinElmer, USA) scanning. A cylindrical standardized region of interest of 5 mm in diameter was used, and data on bone volume/total volume (BV/TV) were obtained using the system software.

After micro-CT scanning, specimens were fixed in 4% paraformaldehyde for 24 h and decalcified in 10% EDTA with 0.1 M PBS for 1 month. Then, the specimens were stained with H&E, Masson's trichrome (Masson), collagen type I (Col I), CD68, iNOS, CD206, OCN, and CD31.

### Statistical analysis

All experiments were performed in triplicate, unless otherwise indicated. Data were expressed as means ± standard deviations (SDs). Statistical analysis was performed using SPSS Statistics 16.0 (Chicago, IL, USA) using one-way analysis of variance, followed by the Tukey's multiple-comparison test to evaluate between-group differences. *p* < 0.05 was considered statistically significant.

## Results

### General observation, structure, and physical properties of scaffolds

**Fig. 1:**
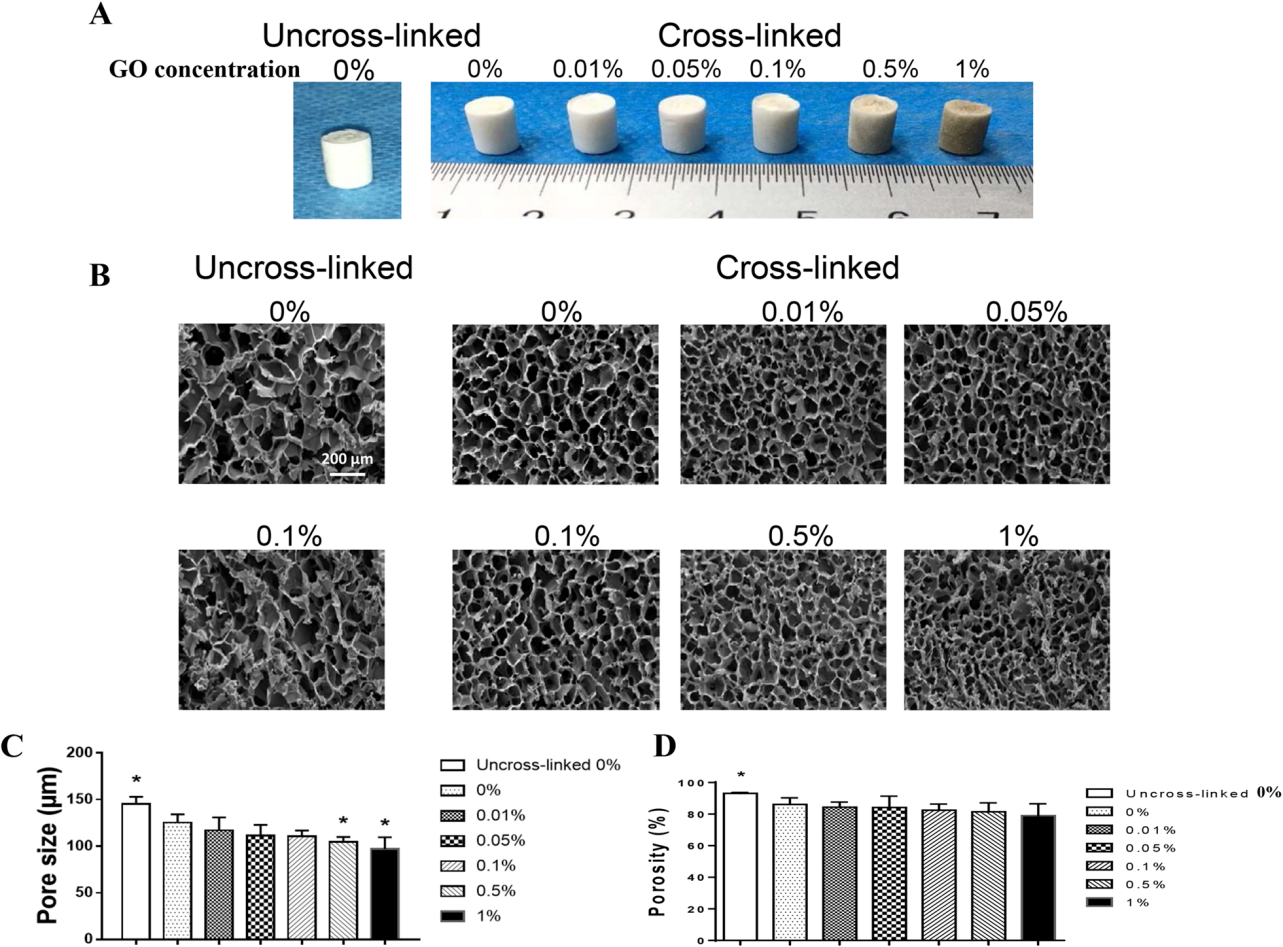
**a** Macroscopic images of the scaffolds containing various concentrations of GO before or after cross-linking. **b** SEM images of the scaffolds. Scale bar = 200 μm. **c** Average pore size of each scaffold. **d** Porosity of each scaffold

The GO sheets after dispersion appeared as 2D single-layer flakes with a mean lateral size of 291.9 ± 171.9 nm (Additional file 1: Figure S1). The C/O ratio was ~ 2.21. The scaffolds were prepared successfully, and the general observations are shown in Fig. 1a. The scaffolds without GO were white and spongelike, and scaffolds containing GO were gray. The color of the scaffold became darker as the concentration of GO increased. SEM results showed that the cross-linked scaffolds had a more regular internal structure compared with the uncross-linked scaffolds (Fig. 1b). The cross-linked scaffolds also possessed more interconnected pores. The uncross-linked 0% group had the largest average pore size (145.18 ± 7.94 μm) (Fig. 1c). The pore diameter decreased with the increase in GO concentration (0%, 125.27 ± 8.94 μm; 0.1%, 110.34 ± 6.49 μm; 1%, 96.94 ± 12.66 μm). Similar results were observed in the porosity test, but the porosity of each group exceeded 80% except for the 1% group (Fig. 1d). These results indicated that the scaffolds with cross-linking had orderly and interconnected microstructures that could be conducive to nutrient exchange and cell growth.

**Fig. 2:**
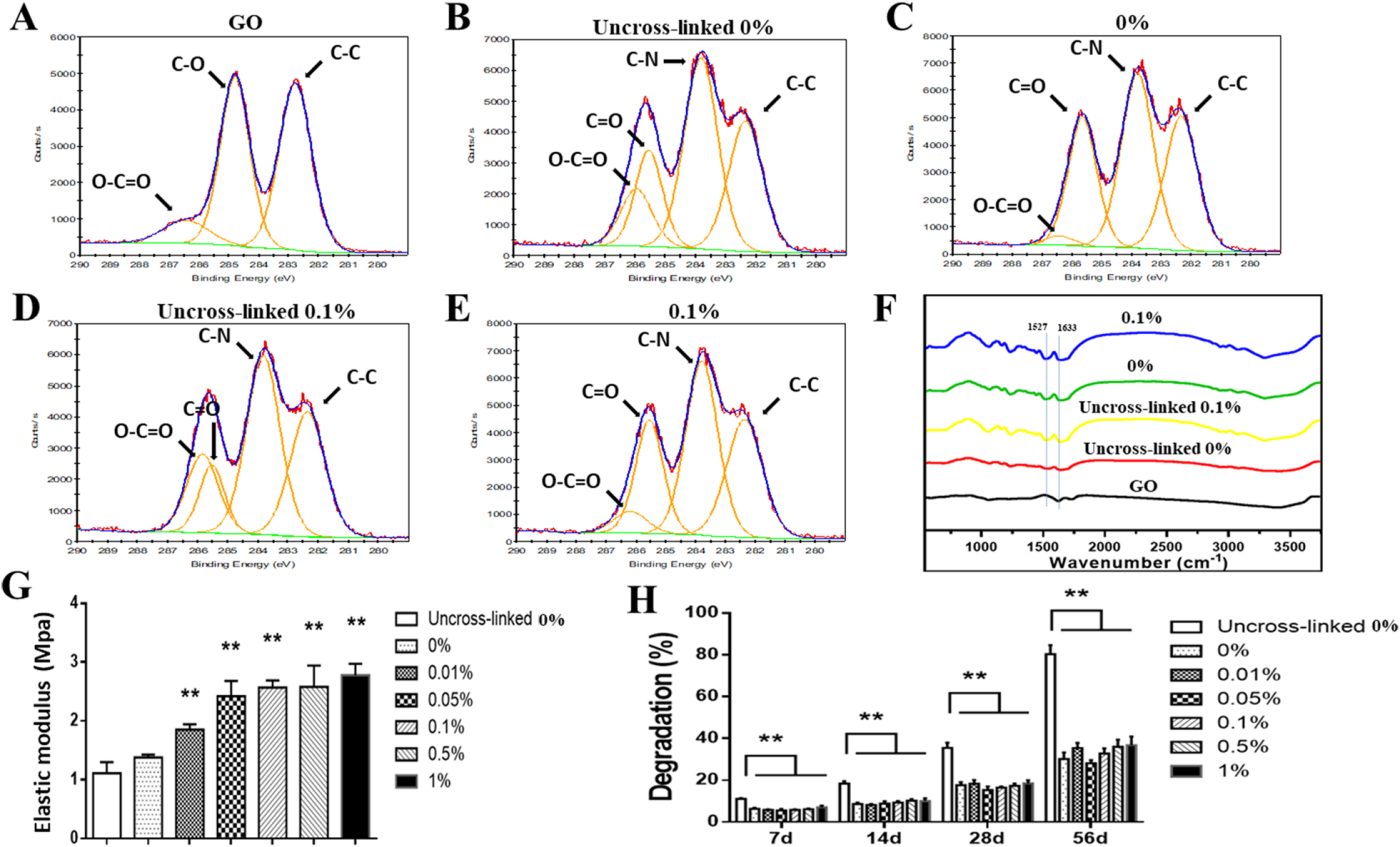
**a**–**e** XPS analyses of GO powder, the uncross-linked 0%, 0%, uncross-linked 0.1%, and 0.1% groups. Scaffolds containing 0.1% GO before and after cross-linking are labeled “Uncross-linked 0.1%” and “0.1%,” respectively. Scaffolds without GO before and after cross-linking are labeled “Uncross-linked 0%” and “0%,” respectively. **f** FTIR spectra of the scaffolds. **g** Compressive elastic modulus of each scaffold. **h** Degradation of each scaffold. Statistically significant differences are indicated with *p < 0.05 and **p < 0.01 versus the 0% group

The XPS results showed that the O–C=O peak in the uncross-linked groups was less intense compared with the cross-linked groups, and there was more C–N bond formation in the cross-linked groups, indicating a successful amidation reaction in cross-linking (Fig. 2a–e) (Additional file 1: Table S1). The FTIR examination showed a similar result—the peaks at 1633 cm^−1^ (amide I) and 1527 cm^−1^ (amide II) were obvious in the cross-linked groups (Fig. 2f). However, the uncross-linked 0.1% group also presented intense peaks at 1633 and 1527 cm^−1^, which might be caused by the benzene ring-like structure (1430–1650 cm^−1^) of GO.

The scaffolds with cross-linking had a higher compressive modulus of elasticity compared with the uncross-linked scaffolds (Fig. 2g). The elastic modulus of the cross-linked scaffolds increased with the increase in GO concentration. The elastic modulus of the 1% group (2.78 ± 0.19 MPa) was about twice that of the 0% group (1.38 ± 0.05 MPa). The cross-linking reaction significantly reduced the degradation rate of the scaffolds. However, there was no significant correlation between the degradation rate and the GO concentration (Fig. 2h). The results of the scaffold characterization indicated that the scaffolds with cross-linking had an ideal structure and enhanced physical properties. Therefore, the scaffolds with cross-linking were used for the subsequent in vitro experiments.

### Cell viability, adhesion, proliferation, and osteogenesis

**Fig. 3:**
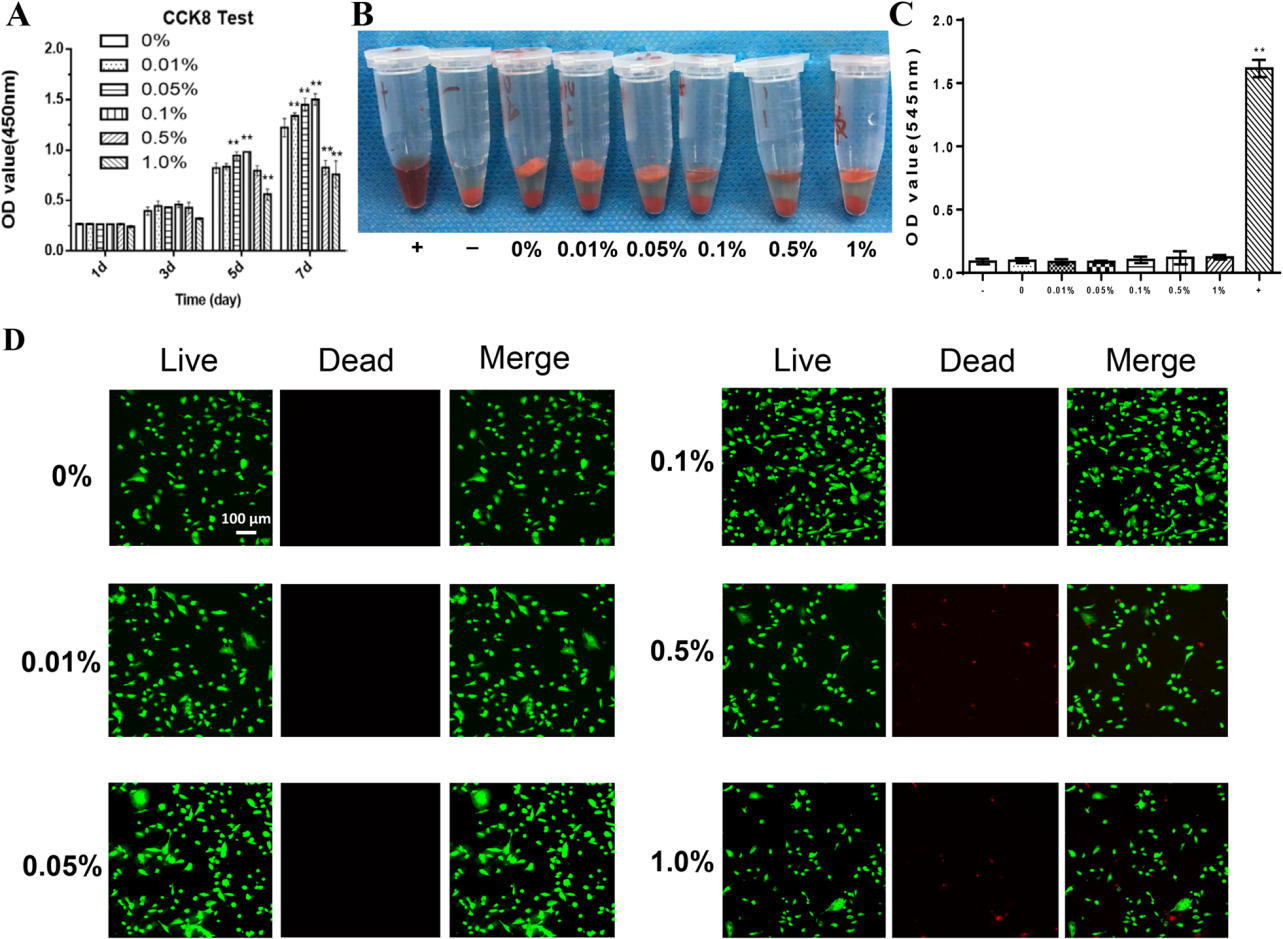
**a** Cytotoxicity assay of USCs cultured with the extraction medium using CCK-8 test. The cross-linked scaffolds containing 0%, 0.01%, 0.05%, 0.1%. 0.5%, and 1% GO were labeled “0%,” “0.01%,” “0.05%,” “0.1%,” “0.5%,” and “1%.” **b** and **c** Hemolysis test of each scaffold. **d** Live–dead staining images of USCs cultured on scaffold for 7 days (green representing living cells and red representing dead cells, scale bar = 100 μm). Statistically significant differences are indicated with *p < 0.05 and **p < 0.01 versus the 0% group

**Fig. 4:**
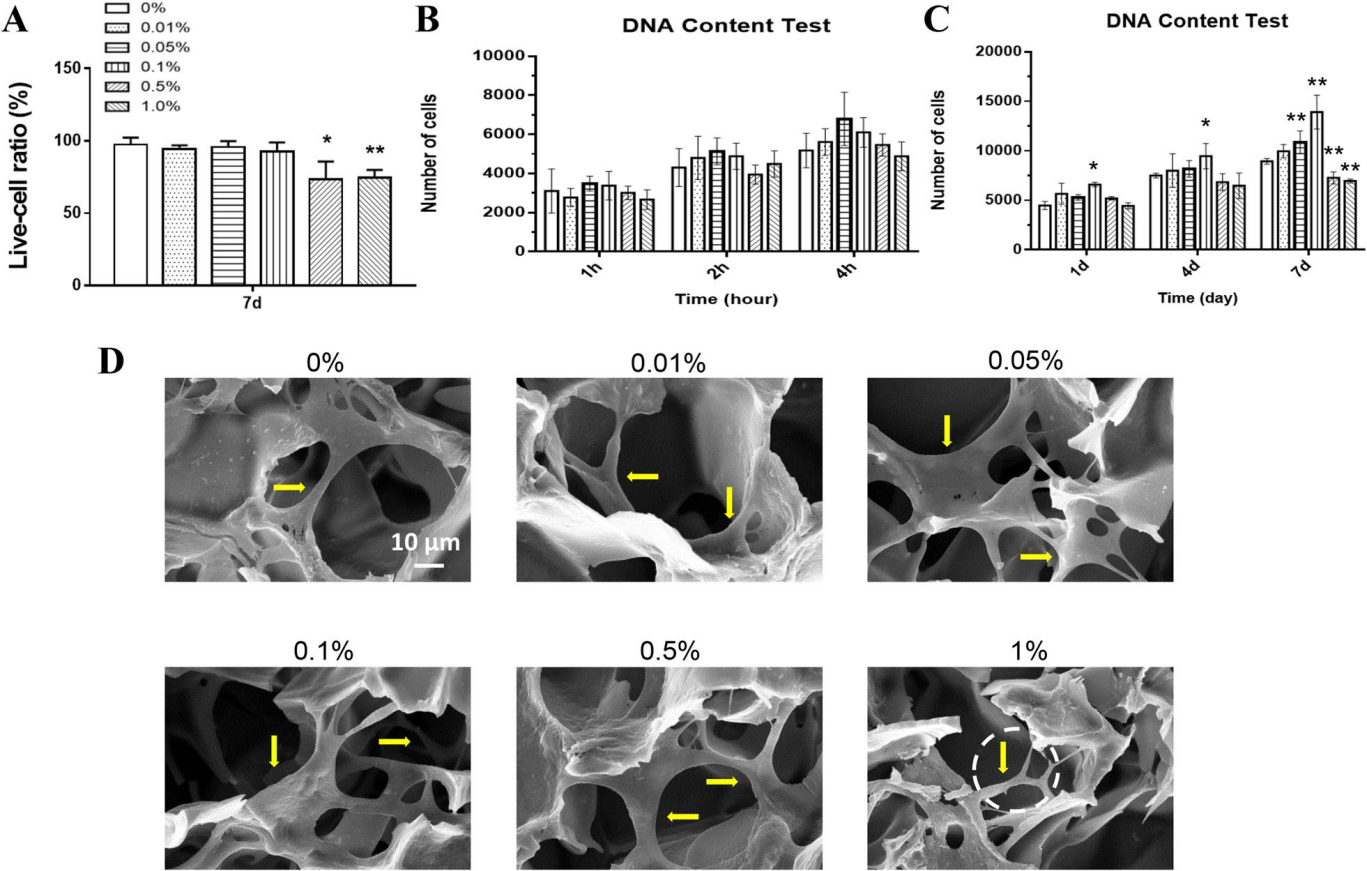
**a** Live cell ratio in live–dead staining results. **b** Cell adhesion test of USCs adhered to each scaffold using the detection of DNA content. The cross-linked scaffolds containing 0%, 0.01%, 0.05%, 0.1%. 0.5%, and 1% GO were labeled “0%,” “0.01%,” “0.05%,” “0.1%,” “0.5%,” and “1%.” **c** Cell proliferation test of USCs cultured on each scaffold using the detection of DNA content. **d** SEM images of USCs adhered to each scaffold after 7 days of culture. Scale bar = 10 µm. Statistically significant differences are indicated with *p < 0.05 and **p < 0.01 versus the 0% group

The biocompatibility of the scaffolds was evaluated using a CCK-8 test, hemolysis assay, and live–dead staining. For the CCK-8 test, the scaffolds' extraction medium was used to detect the cytotoxicity. From Day 1 to Day 3, no significant difference was found in the cell numbers among the six groups (Fig. 3a). On Day 7, the cell numbers in the 0.5% and 1% groups were significantly reduced compared with the other groups. The results of hemolysis assay showed that there was no obvious hemolysis in all the groups (Fig. 3b, c). The live–dead staining results showed that after the USCs were cultured on each scaffold for 7 days, the number of living cells decreased in the 0.5% and 1% groups compared with the other groups, although most of the cells on each scaffold were alive (Fig. 3d). And the live-cell ratios in the 0.5% and 1% groups also decreased significantly compared with the other groups (Fig. 4a). These results indicated that the scaffolds with GO concentrations less than 0.5% had good biocompatibility.

As shown in Fig. 4a, all six groups had a similar adhesion rate after 4 h of incubation. The proliferation rate of USCs in the 0.05% and 0.1% groups was significantly higher than that in the 0% group, and the cell number in the 0.5% and 1% groups significantly decreased compared with the 0% group (Fig. 4b). SEM results showed that USCs could adhere to and grow into each scaffold after 7 days of culture. The cells in each group except the 1% group presented a well-spread shape with lamellipodia/filopodia extending into the scaffolds (Fig. 4c). These results suggested that the 0.05% and 0.1% groups promoted cell proliferation, and the scaffolds containing less than 1% GO endowed the adhered cells with an ideal morphology for cell growth.

**Fig. 5:**
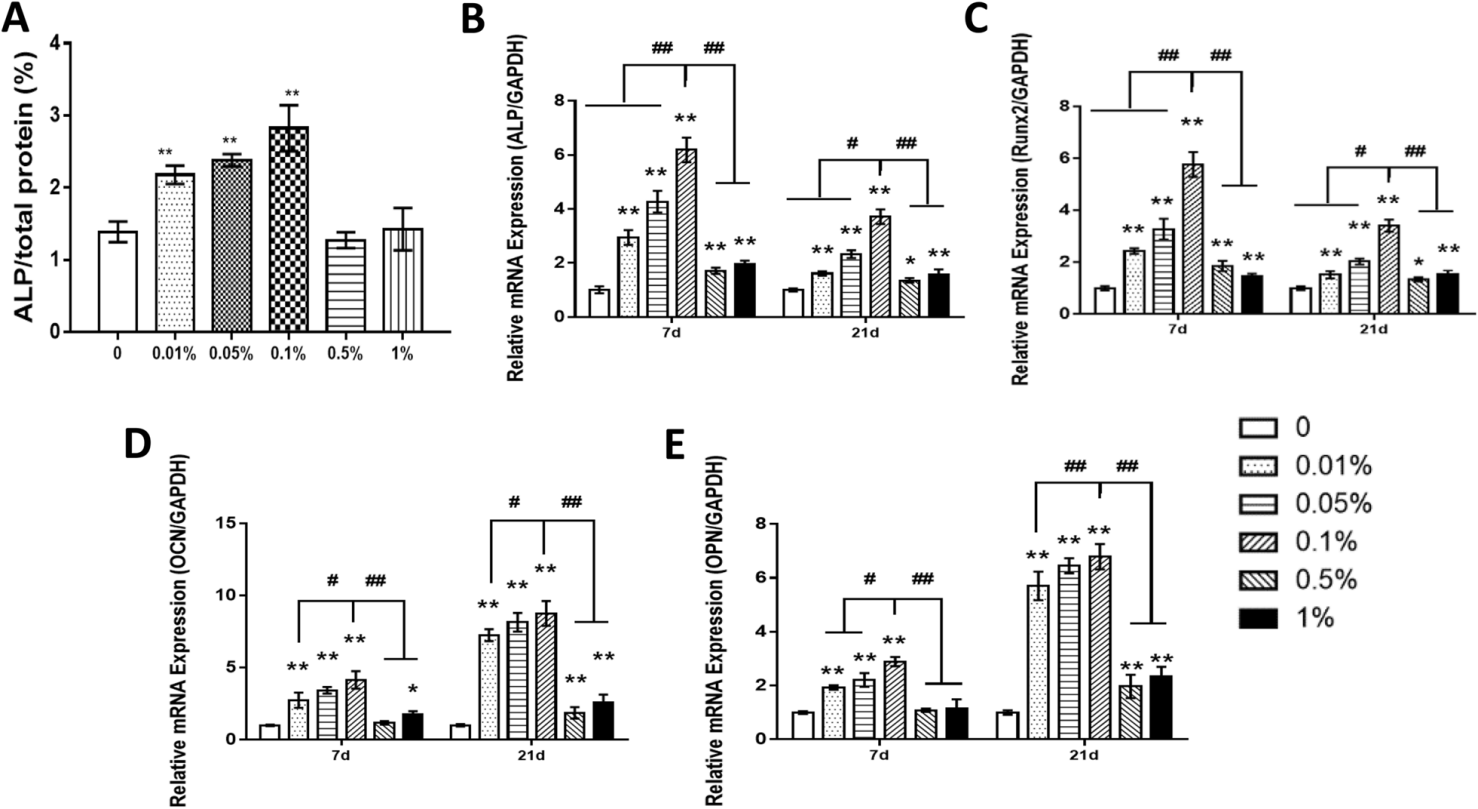
**a** Normalized ALP activity detected at 10 days of osteogenic induction of USCs cultured on each scaffold. The cross-linked scaffolds containing 0%, 0.01%, 0.05%, 0.1%. 0.5%, and 1% GO were labeled “0%,” “0.01%,” “0.05%,” “0.1%,” “0.5%,” and “1%.” **b**–**e** Detection of the mRNA levels of selected osteogenic markers in USCs at 7 and 21 days of osteogenic induction. *p < 0.05 and **p < 0.01 versus the 0% group. #*p* < 0.05 and ##*p* < 0.01 versus the 0.1% group

After 10 days of osteogenic induction, the ALP activity in the 0.01%, 0.05%, and 0.1% groups was higher than that in the other groups, and the 0.1% group showed the highest expression of ALP activity (Fig. 5a). The osteogenesis-related genes of the USCs were significantly upregulated on the scaffolds with GO concentrations less than 0.5% at both 7 and 14 days (Fig. 5b–e). The results of the aforementioned in vitro experiments indicated that the scaffold containing 0.1% GO had the strongest ability to promote cell proliferation and osteogenic differentiation. Therefore, the scaffolds containing 0.1% GO were used in the follow-up experiments.

### Induction of M2-type differentiation of macrophages via scaffolds loaded with USCs

**Fig. 6:**
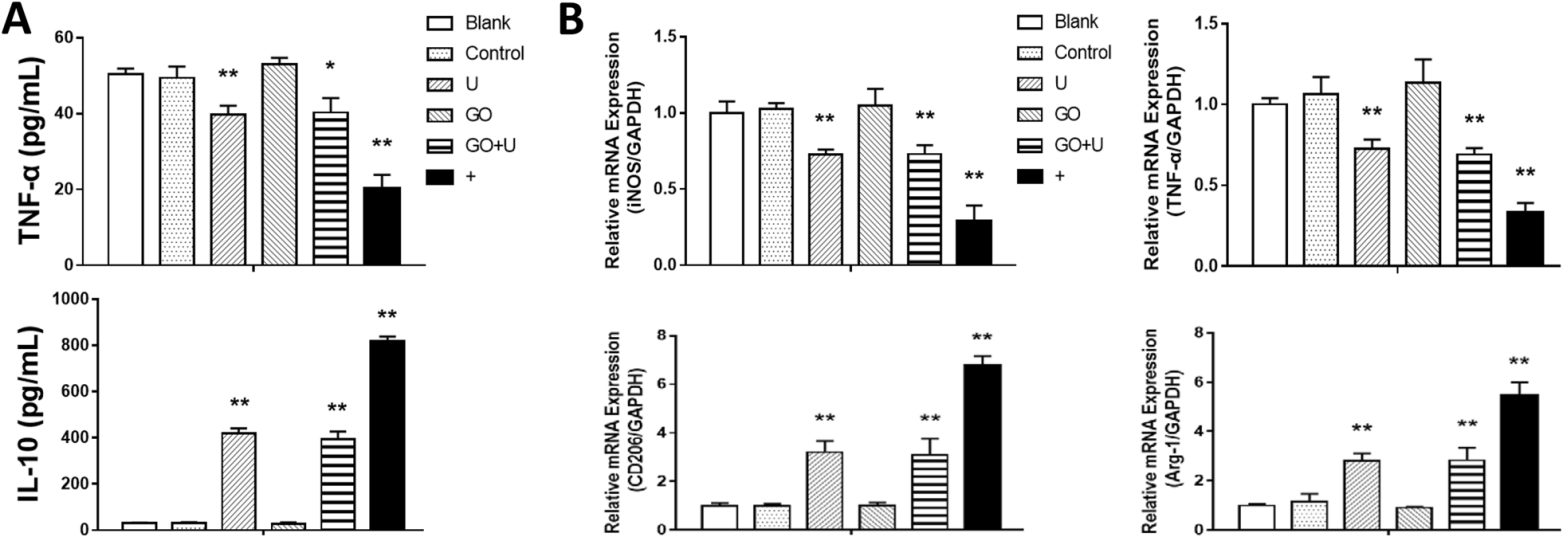
**a** TNF-α and IL-10 expressions in the supernatant after the co-culture of macrophages and scaffolds for 48 h. The scaffolds without GO were labeled “control,” the GO-free scaffolds loaded with 1.5 × 10^5^ USCs were labeled “U,” the scaffolds containing 0.1% GO were labeled “GO,” and the scaffolds containing 0.1% GO loaded with 1.5 × 10^5^ USCs were labeled “GO+U.” Macrophages cultured with the induction medium for M2-type macrophages were used as a positive control (+), and macrophages cultured with only culture medium were labeled “Blank.” **b** Detection of the mRNA levels of selected genes related to M1- and M2-type macrophage after the co-culture of macrophages and scaffolds for 48 h. Statistically significant differences are indicated with *p < 0.05 and **p < 0.01 versus the control group

**Fig. 7:**
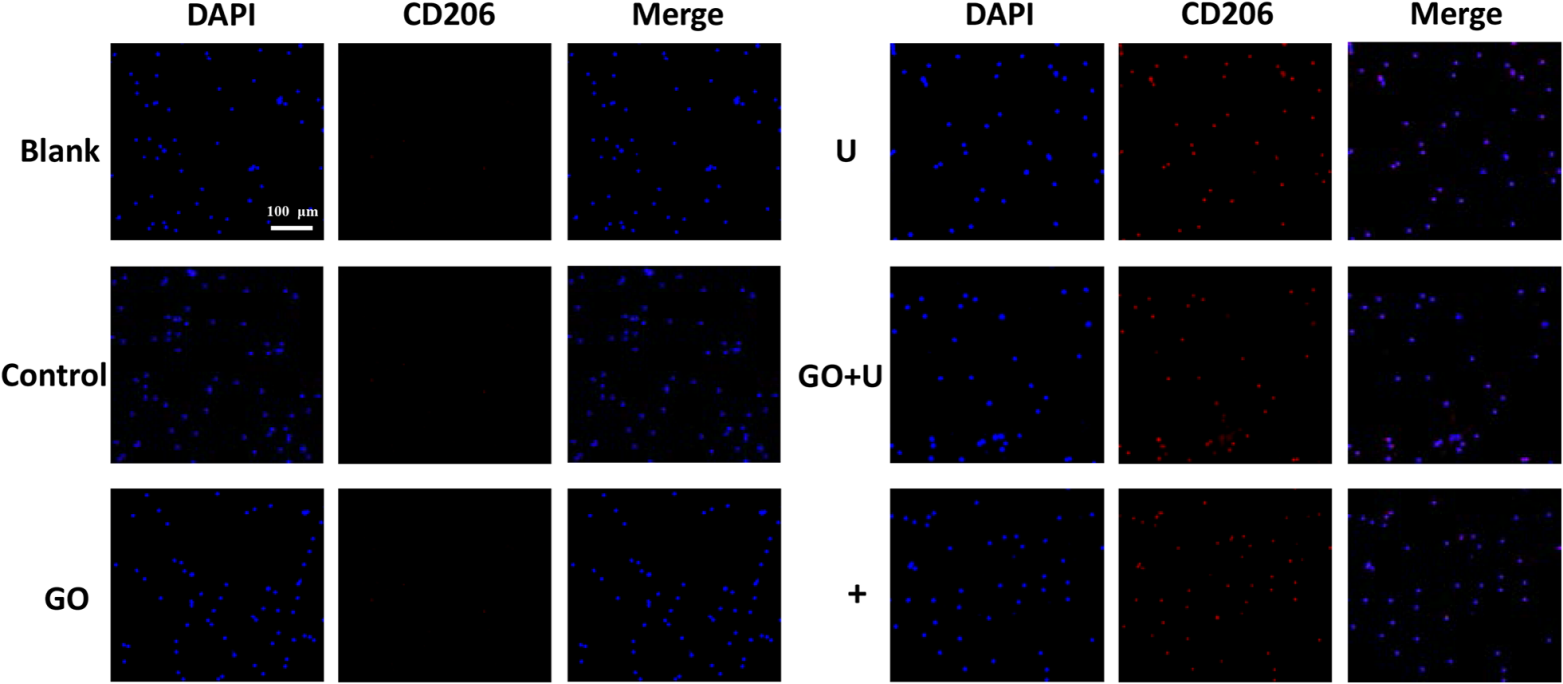
Images of CD206 immunofluorescence staining of macrophages co-cultured with each scaffold for 48 h. Scale bar = 100 μm. The scaffolds without GO were labeled “control,” the GO-free scaffolds loaded with 1.5 × 10^5^ USCs were labeled “U,” the scaffolds containing 0.1% GO were labeled “GO,” and the scaffolds containing 0.1% GO loaded with 1.5 × 10^5^ USCs were labeled “GO+U.” Macrophages cultured with the induction medium for M2-type macrophages were used as a positive control (+), and macrophages cultured with only culture medium were labeled “Blank”

To evaluate the effect of the scaffold on macrophage differentiation, Elisa and qRT-PCR were used to test the involved proteins and genes. After 2 days of coculture, the expression level of TNF-α in the supernatant of the U, GO+U, and positive control (+) groups was significantly lower than that in the Blank, control, and GO groups. The IL-10 expression in the U, GO+U, and positive control groups was much higher than that in the other groups (Fig. 6a). The tests of qRT-PCR presented similar results, showing that the markers of the M1 phenotype (*iNOS* and *TNF-α*) were less expressed in the U, GO+U, and positive control groups, and the markers of the M2 phenotype (CD206 and Arg-1) were highly expressed in these three groups (Fig. 6b). The images of CD206 immunofluorescence staining showed that the number of positive cells was far greater in the U, GO+U, and positive control groups than in the other groups (Fig. 7). These results suggested that the scaffolds loaded with USCs inhibited the M1-type differentiation of the macrophages and promoted the M2-type differentiation.

### Inflammatory response of scaffolds after subcutaneous implantation

**Fig. 8:**
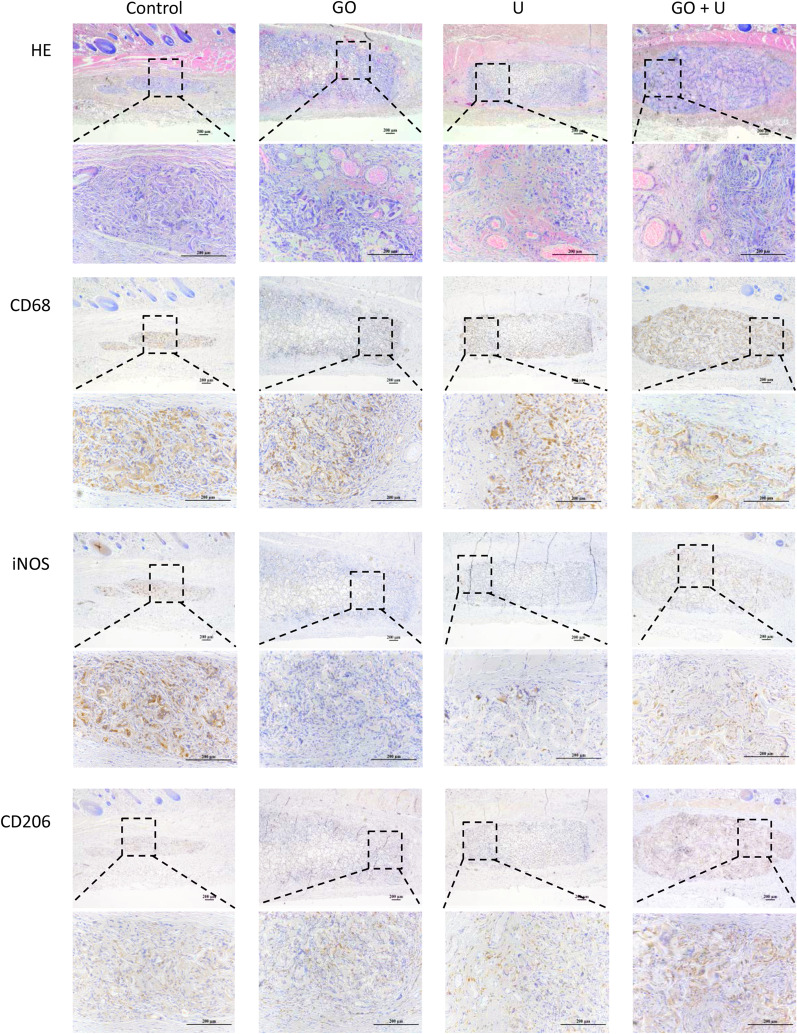
Histological evaluations for inflammatory response of scaffolds at 7 days of subcutaneous implantation, including H&E, CD68, iNOS, and CD206 staining. The scaffolds without GO were labeled “control,” the GO-free scaffolds loaded with 5 × 10^5^ USCs were labeled “U,” the scaffolds containing 0.1% GO were labeled “GO,” and the scaffolds containing 0.1% GO loaded with 5 × 10^5^ USCs were labeled “GO+U.” Scale bar = 200 μm

**Fig. 9:**
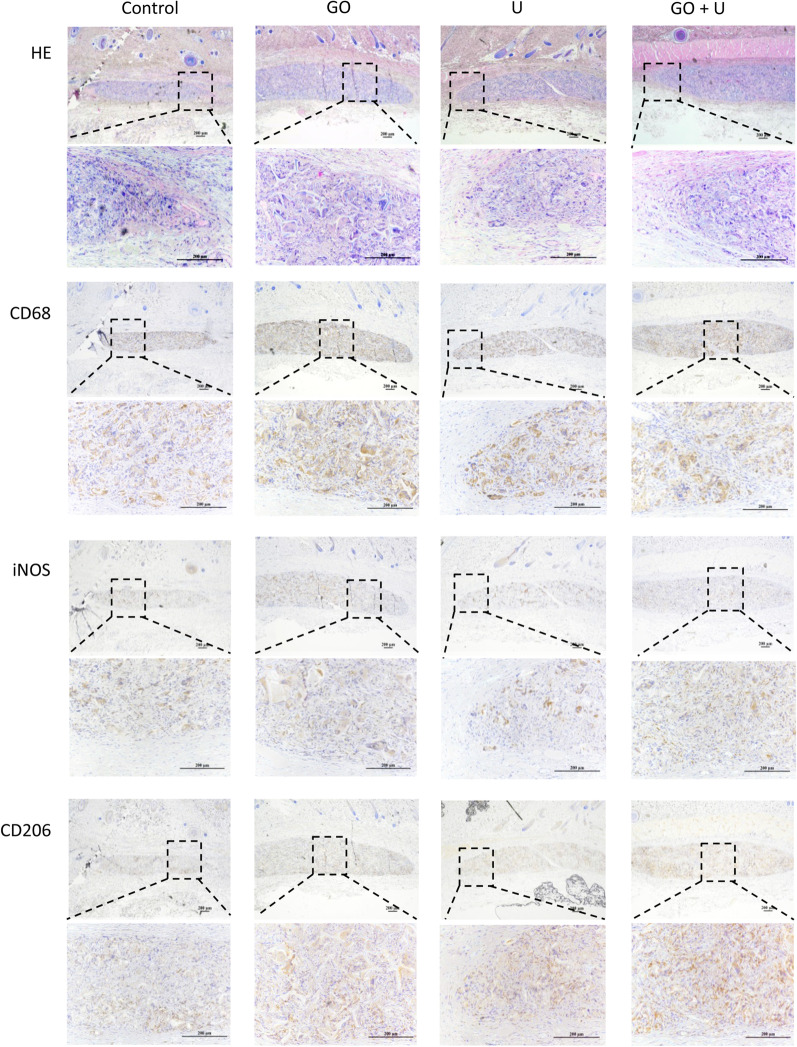
Histological evaluations for inflammatory response of scaffolds at 14 days of subcutaneous implantation, including H&E, CD68, iNOS, and CD206 staining. The scaffolds without GO were labeled “control,” the GO-free scaffolds loaded with 5 × 10^5^ USCs were labeled “U,” the scaffolds containing 0.1% GO were labeled “GO,” and the scaffolds containing 0.1% GO loaded with 5 × 10^5^ USCs were labeled “GO+U.” Scale bar = 200 μm

The degradation and inflammatory reaction of the scaffolds were evaluated using the subcutaneous implantation test in rats. At 3 days postsurgery, all the scaffolds caused mild inflammation and were infiltrated by a small number of macrophages (Additional file 1: Figure S2). At 7 days of implantation, the scaffold in the control group was degraded significantly, and the degradation rate of the GO group was the lowest (Fig. 8). There were large numbers of macrophages in each group. The macrophages in the control group were mainly stained with iNOS, and CD206 staining was more intense in the other groups. All the scaffolds were degraded obviously at 14 days postsurgery (Fig. 9). Most of the macrophages were stained with CD206 in each group, and a higher rate of positive staining of CD206 was observed in the U and GO+U groups. These results indicated that both the incorporation of GO and USCs reduced the inflammatory response in the early stage of implantation-induced inflammation via the inhibition of the M1-type differentiation of macrophages. Furthermore, scaffolds loaded with USCs promoted the M2-type differentiation of macrophages in the middle and late stages of biomaterial-induced inflammation, which could accelerate tissue repair.

### In vivo osteogenesis

**Fig. 10:**
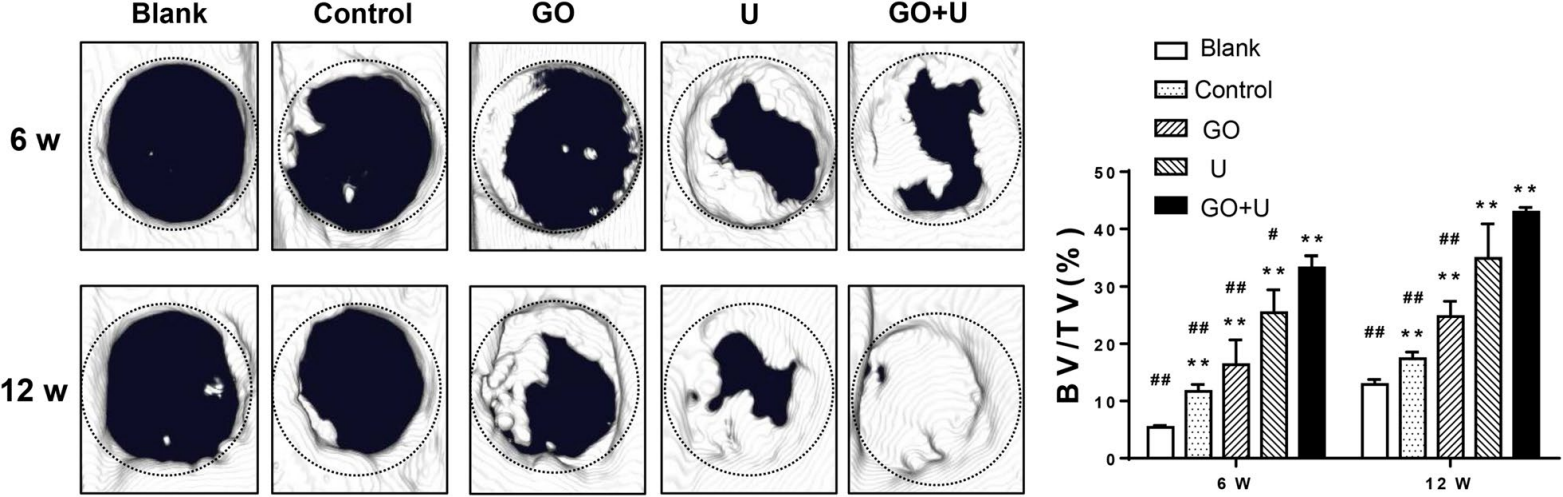
Micro-CT results of each group. BV/TV represents bone volume to total volume. The scaffolds without GO were labeled “control,” the GO-free scaffolds loaded with 5 × 10^5^ USCs were labeled “U,” the scaffolds containing 0.1% GO were labeled “GO,” and the scaffolds containing 0.1% GO loaded with 5 × 10^5^ USCs were labeled “GO+U.” The rats without a scaffold implantation were labeled “Blank.” *p < 0.05 and **p < 0.01 versus the blank group. #*p* < 0.05 and ##*p* < 0.01 versus the GO+U group

Cranial bone defects in rats were created to verify the ability of the scaffolds to facilitate bone repair. The results of the micro-CTs showed that new bone formed from the edge toward the center of the implanted area, and the new bone volume in the GO+U group was the highest at 6 and 12 weeks of implantation, followed by those in the U and GO groups (Fig. 10). The control group exhibited the poorest bone repair effect.

**Fig. 11:**
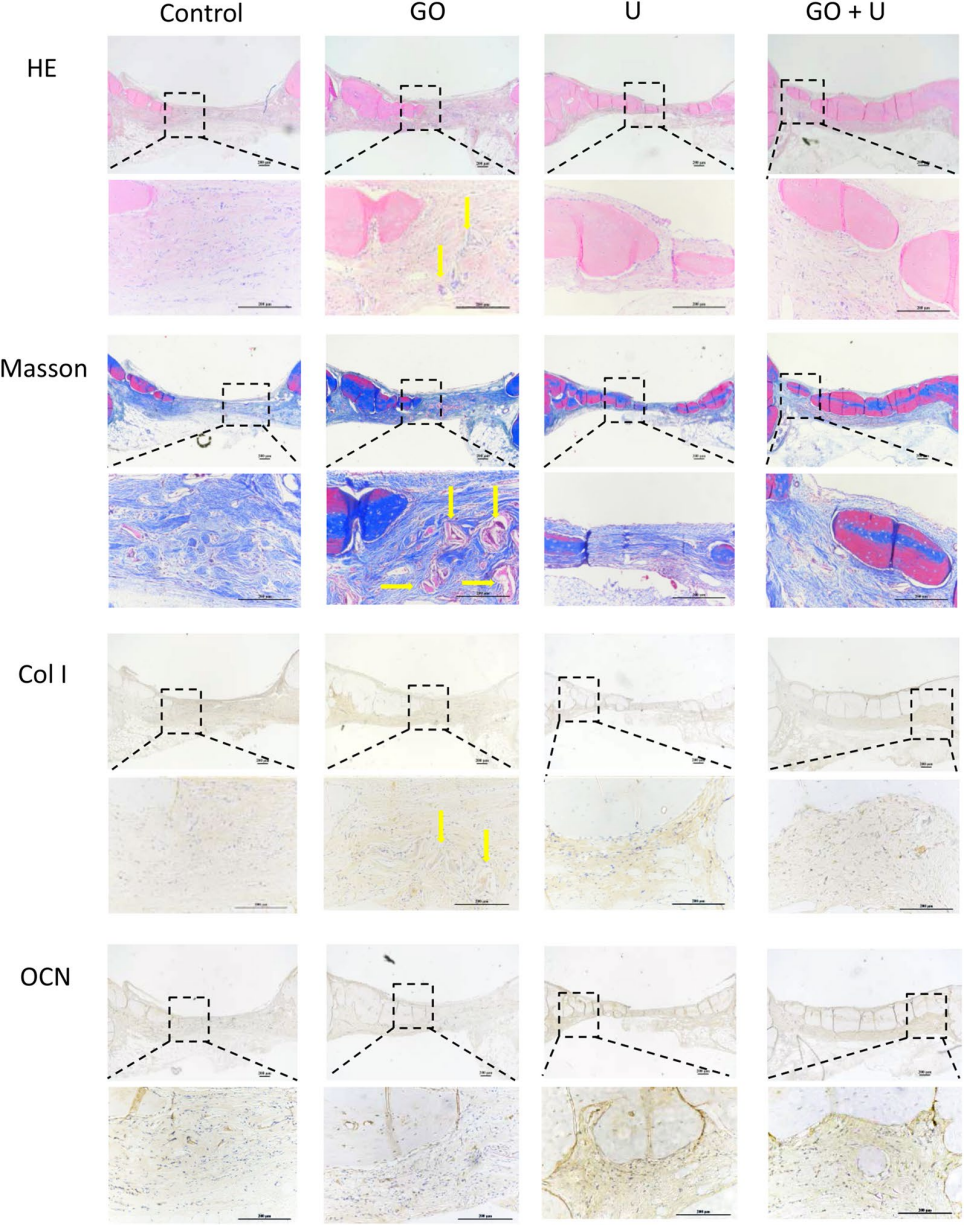
H&E, Masson, Col I, and OCN staining images of the specimens from each group after 12 weeks of scaffold implantation. The scaffolds without GO were labeled “control,” the GO-free scaffolds loaded with 5 × 10^5^ USCs were labeled “U,” the scaffolds containing 0.1% GO were labeled “GO,” and the scaffolds containing 0.1% GO loaded with 5 × 10^5^ USCs were labeled “GO+U.” Scale bar = 200 μm. Yellow arrows represent the remnant SF

**Fig. 12:**
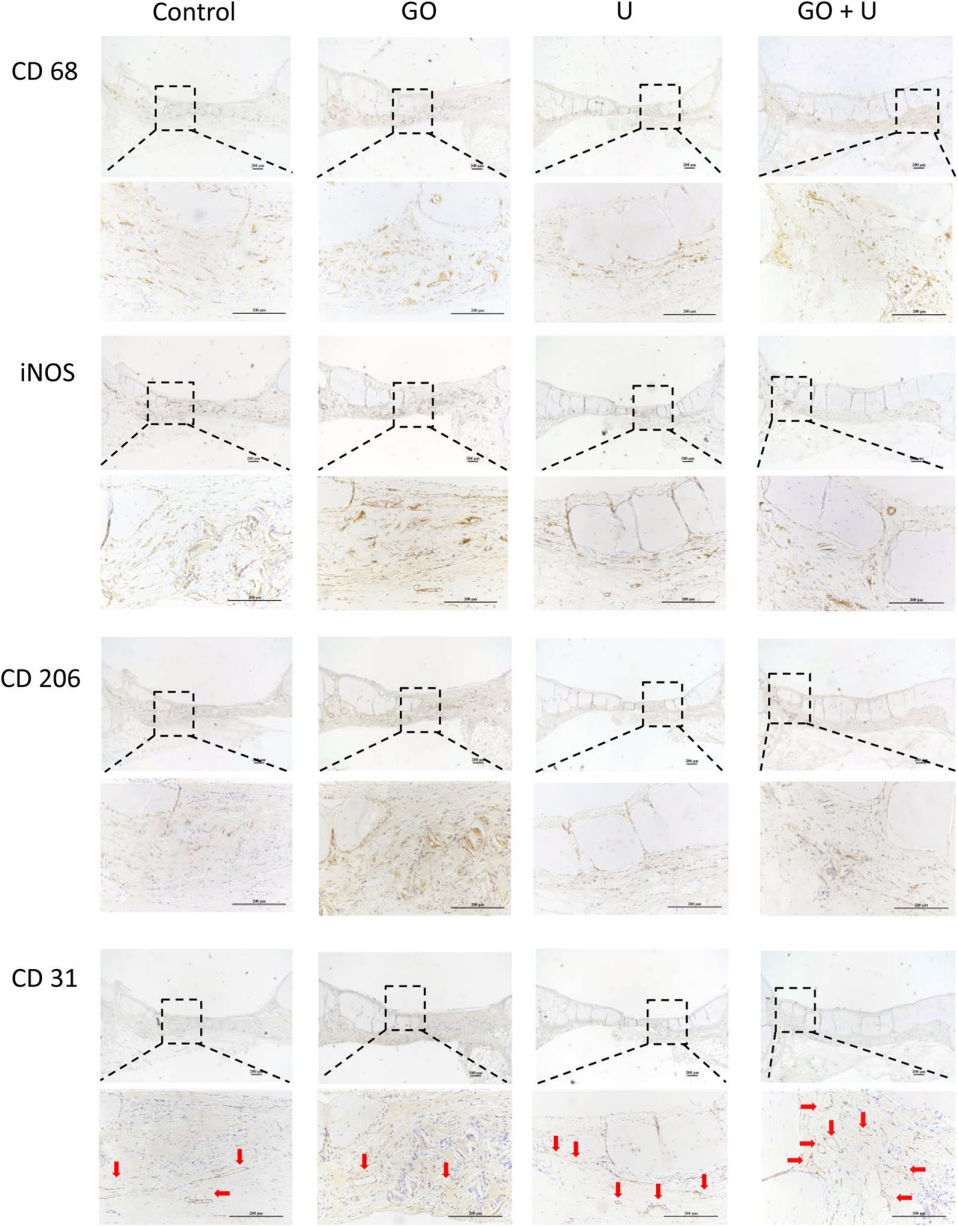
Images of CD68, iNOS, CD206, and CD31 staining of specimens after 12 weeks of scaffold implantation. The scaffolds without GO were labeled “control,” the GO-free scaffolds loaded with 5 × 10^5^ USCs were labeled “U,” the scaffolds containing 0.1% GO were labeled “GO,” and the scaffolds containing 0.1% GO loaded with 5 × 10^5^ USCs were labeled “GO+U.” Scale bar = 200 μm. Red arrows represent new vessels

Histological staining was also performed to evaluate the bone repair effect of each scaffold. At 6 weeks postimplantation, there was a large amount of collagen formation in the control, U, and GO+U groups. The collagen was mainly composed of Col I in the U and GO+U groups, while that in the control group was rarely stained with Col I (Additional file 1: Figure S3). The GO+U group presented obvious positive staining of OCN and CD31 compared with the other groups (Additional file 1: Figure S4). These results suggested that the GO+U group had the strongest potential for bone regeneration and vessel formation. At 12 weeks of implantation, the new bone in the GO+U group almost bridged the injury site and was the thickest among the four groups (Fig. 11). In addition, this group still exhibited intense positive staining of Col I and CD31 (Figs. 11, 12). A low expression of OCN was observed in the GO+U group at 12 weeks postsurgery compared with that at 6 weeks, which might be caused by the maturation of new bones.

Immunohistochemical staining of inflammation-related indicators was performed to assess the immunomodulation effects of scaffolds on osteogenesis. At 6 weeks postimplantation, the CD206 staining was more intense in the U and GO+U groups than in the control and GO groups (Additional file 1: Figure S4), suggesting a higher proportion of M2-type macrophages in the U and GO+U groups. The high proportion of M2-type macrophages could accelerate bone regeneration and induce a better bone repair effect. At 12 weeks of implantation, the number of macrophages decreased in the U and GO+U groups, and the tissue repair entered the end stage (Fig. 12). The number of M1-type macrophages still remained at high levels in the Control and GO groups, and this long-term inflammatory response seemed to affect bone repair. These results indicated that the scaffolds loaded with USCs could promote M2-type differentiation of macrophages and enhance the ultimate bone repair effect. The USC-laden scaffolds containing 0.1% GO exhibited the best capacity for immunomodulation and accelerating bone regeneration.

## Discussion

Tissue-engineered bone is considered to be one of the most ideal materials used to treat bone defects. SCs are often used as seeding cells in traditional tissue engineering. The shortcomings of SCs, including invasive isolation procedures and the scarcity of sources, limit the clinical applications of cell-based tissue engineering using SCs [10, 11]. On the other hand, cell-free tissue engineering is usually expensive and involves a complicated preparation process that also hinders the clinical transformation of cell-free tissue-engineered bones. In order to avoid the limitations of traditional SCs and most cell-free tissue-engineered bones, USCs were used in this study as we hoped to provide a promising platform for stem cell-based tissue-engineered bones. There are many advantages to using USCs including the ease and noninvasiveness of their isolation, their fast and stable proliferation, and the capacity to repeatedly isolate them from the same individual [44]. USCs are derived from kidneys and are a reliable source of SCs. Even the USCs obtained from a patient with bladder cancer were not contaminated by tumor cells [45]. In addition, USCs maintain a higher telomerase activity and longer telomere length compared with BMSCs and can proliferate stably for more than 20 passages [46]. USCs exhibited a faster proliferation rate compared with BMSCs and ADSCs [26, 27, 47]. However, USCs showed a weaker osteogenic capacity compared with traditional SCs. Therefore, it was necessary to optimize the scaffold design in order to enhance the USCs' osteogenic ability. Furthermore, USCs presented two phenotypical subpopulations, one rice-like shape and one spindle shape, and the spindle-shaped USCs had a greater capacity for osteogenic differentiation [48]. The screening of USCs’ subpopulations can also be a method to strengthen the osteogenic ability of USCs in the future. In this study, a GO-modified SF/nHA scaffold was developed to load with USCs and improve the osteogenesis-related behaviors of USCs for bone repair.

GO modification has been demonstrated to enhance the physical properties of various biomaterials [49, 50]. In the present study, scaffolds with GO showed more orderly and interconnected microstructures and slower degradation rates that were conducive to the exchange of liquids and nutrients and provided a sustained scaffold function for repair-related cells [51, 52]. The mean pore size of each scaffold was more than 100 μm, which could provide enough space for the exchange of nutrients and wastes, vascularization, and osteogenesis-related cell ingrowth [53–55]. Furthermore, the modification of GO also enhanced the elastic modulus of the scaffolds, which could have helped to maintain the physical form of the scaffolds. The mechanism of GO’s promotion of cell proliferation and differentiation may include two aspects: On one hand, GO can enrich local nutrients including GFs and keep them at a high concentration; on the other hand, GO can participate in the activation of related signaling pathways, such as the calmodulin-kinases, MAPK, and Erk1/2 pathways [56–59]. When cells are seeded onto scaffolds, GO in the surface will be in direct contact with the cells. Cell adhesion and morphology will be modulated via the signaling pathways. GO of appropriate concentration can also promote cell growth and differentiation [60]. Some of the cells will gradually grow into the pores, and GO within the porous structure will be in further contact with these cells. The GO in the deep part of the scaffold may mainly act as the role of enriching the local nutrients or growth factors for the modulation of cell fate. In addition, after scaffold implantation, the GO in the surface can also provide a supportive milieu for angiogenesis, inflammatory modulation, and metabolism stabilization [53, 61]. However, the biotoxicity of the scaffolds increases with an increase in the concentration of GO. The scaffolds containing more than 0.5% GO inhibited USC proliferation and affected the morphology of the adhered cells. This is because the direct contact between GO and the cell membrane may have induced oxidative stress and produced reactive oxygen species (ROS) [62]. The high level of ROS can affect cell growth and cause apoptosis [63]. In addition to GO modification, the concentration of SF was also downregulated to 6% to endow the scaffold with an ideal degradation rate. As we reported before, the SF/nHA scaffolds containing 10% SF were not completely degraded at 12 weeks postimplantation [13]. In the present study, GO modification could further decrease the degradation rate, which would have affected new bone formation. Therefore, we adjusted the SF concentration to make the scaffold have an appropriate degradation rate that matched the bone regeneration rate.

The biomaterial-induced local immune response, especially inflammation, also plays an important role in the final tissue repair effect [17, 64]. M1-type macrophages can secrete proinflammatory cytokines to deal with foreign pathogens, but a large number of M1-type macrophages and a severe inflammatory response will inhibit tissue regeneration [65]. M2-type macrophages can secrete antiinflammatory cytokines including various GFs to alleviate inflammation and promote tissue regeneration [66]. Therefore, suppressing the M1-type differentiation of macrophages and promoting the M2-type differentiation in the middle and late stages of biomaterial-induced inflammation are conducive to tissue repair. SCs’ implantation has been proved to reduce local inflammation and accelerate the M2-type differentiation rate, which can contribute to an enhanced ultimate effect of tissue repair [14, 65]. Indeed, USCs showed greater immunomodulatory capacities compared with BMSCs [46]. In this study, the USC-laden scaffolds promoted the M2-type differentiation of macrophages in both in vitro and in vivo experiments. The GO+U group induced a higher M2-type differentiation rate at 7 and 14 days of subcutaneous implantation and at 6 weeks after the implantation of cranial bone defects. The GO+U group also exhibited the best bone repair effect. In addition to USCs, the GO group also inhibited M1-type differentiation in the subcutaneous implantation experiments. However, this phenomenon was not observed in the in vitro coculture experiment. The difference between the in vitro and in vivo results may be because the macrophages were not in direct contact with the scaffolds in the in vitro experiment. Besides, some researchers believe that GO can promote the M1-type differentiation of macrophages, while others believe that GO can promote the M2-type differentiation [67–69]. These opposing views may be the result of inconsistencies in the GO sheets’ parameters and the macrophages’ source species and tissue/organ source. Moreover, in the early stage of biomaterial-induced inflammation, M1-type macrophages can eliminate pathogens and promote tissue repair-related cells homing, such as stem cells and angiogenesis-related cells [70, 71]. Xue et al. also believed that M1-type macrophages could secrete oncostatin M (OSM) and bone morphogenetic protein-2 (BMP2) for bone regeneration [68]. Therefore, promoting M1-type differentiation of macrophage in the early stage of biomaterial-induced inflammation and promoting M2-type differentiation in the middle and late stages may present better effect on accelerating bone regeneration compared with only M1- or M2-type differentiation. Programmed induction of macrophage differentiation also requires consideration in our future work.

Revascularization is another key element in bone repair. USCs show greater vascularization potential compared with other traditional SCs [27]. Moreover, USCs can secrete VEGF, FGF2, and PDGF to promote angiogenesis [48]. In addition, GO can also promote neovascularization [59, 67]. In the current study, the GO+U group had the most areas of positive CD31 staining, indicating the most newly formed vessels. In conclusion, the USC-laden scaffold containing 0.1% GO possessed the best capacity for immunomodulation, neovascularization, and osteogenesis.

The present study had several limitations, however. First, because of USCs’ low immunogenicity and the consistency of the immunomodulatory comparison between the in vitro and in vivo experiments, rat macrophages were used in this study. The coculture of human macrophages and USCs is required before clinical applications. Second, more angiogenesis-related experiments involving human umbilical vein endothelial cells or vascular endothelial cells should be performed to test the angiogenic ability of the scaffolds. Third, the specific mechanism of the synergistic effect of GO and USCs on immunomodulation and bone regeneration should also be further explored. Finally, an animal model of a weight-bearing bone defect is recommended in future studies. Furthermore, a long-term follow-up of the animals involved in the experiments is required to evaluate their health including tumorigenesis and the safety of the procedures after the implantation of USCs.

## Conclusions

We developed a GO-modified SF/nHA scaffold to load with USCs for immunomodulation and bone repair. The scaffold was cross-linked with EDC/NHS. The cross-linked scaffolds had ideal microstructures and better physical properties compared with the uncross-linked ones. The pore size decreased and the compressive modulus of elasticity increased with an increase in the GO concentration, respectively. The scaffolds containing less than 0.5% GO showed good biocompatibility, promoted USC proliferation, and enhanced USC osteogenesis. The scaffolds containing 0.1% GO exhibited the strongest ability to promote the osteogenic differentiation of the USCs. The results of the coculture of the scaffolds and macrophages and the subcutaneous implantation test showed that the incorporation of GO or USCs induced the M2-type differentiation and inhibited the M1-type differentiation of the macrophages. In the rat cranial defect repair experiments, the USC-laden scaffolds containing 0.1% GO promoted the M2-type differentiation of the macrophages in the middle and late stages of biomaterial-induced inflammation and almost bridged the injury site at 12 weeks postsurgery. This GO-modified scaffold loaded with USCs may comprise a powerful platform for tissue-engineered bones to treat clinical bone defects.

## Supplementary Information

**Additional file 1**. **Figure S1.** TEM image of GO after dispersion. Scale bar = 0.5 μm. **Figure S2.** Histological evaluations for inflammatory response of scaffolds after 3 days of subcutaneous implantation, including H&E, CD68, iNOS, and CD206 staining. Scale bar = 200 μm. **Figure S3.** H&E, Masson, and Col I staining images of the specimens from each group at 6 weeks postsurgery. Scale bar = 200 μm. **Figure S4.** Images of CD68, iNOS, CD206, OCN, and CD31 staining of specimens after 6 weeks of implantation. Scale bar = 200 μm. Red arrows represent new vessels. **Table S1.** The percentages of O-C=O and C-N bond in scaffolds.

## Data Availability

The raw data required to reproduce these findings are available on reasonable request from the corresponding author (Z.X.).

## Abbreviations

GFs: Growth factors
SCs: Stem cells
ADSCs: Adipose-derived stem cells
BMSCs: Bone marrow mesenchymal stem cells
iPSCs: Induced pluripotent stem cells
TNF-α: Tumor necrosis factor-α
IL-6: Interleukin-6
IL-10: Interleukin-10
TGF-β: Transforming growth factor-β
USCs: Urine-derived stem cells
GO: Graphene oxide
nHA: Nano-hydroxyapatite
SF: Silk fibroin
cCNF: Carboxylated cellulose nanofibrils
EDC: 1-Ethyl-3-(3-dimethylaminopropyl) carbodiimide hydrochloride
NHS: N-hydroxy succinimide
SEM: Scanning electron microscopy
SBF: Simulated body fluid
FTIR: Fourier transform infrared spectroscopy
XPS: X-ray photoelectron spectrometer
K-SFM: Keratinocyte serum-free medium
DMEM-HG: Dulbecco’s modified Eagle’s medium–high glucose
FBS: Fetal bovine serum
EGF: Epidermal growth factor
BPE: Bovine pituitary extract
CCK-8: Cell counting kit-8
EDTA: Ethylenediaminetetraacetic acid
RBCs: Red blood cells
qRT-PCR: Quantitative reverse transcription polymerase chain reaction
ALP: Alkaline phosphatase activity
Runx2: Runt-related factor-2
OCN: Osteocalcin
Opn: Osteopontin
GAPDH: Glyceraldehyde 3-phosphate dehydrogenase
iNOS: Inducible nitric oxide synthase
Arg-1: Arginase-1
DAPI: 4′,6-Diamidino-2-phenylindole
SD: Sprague–Dawley
H&E: Hematoxylin and eosin
BV/TV: Bone volume/total volume
Masson: Masson's trichrome
Col I: Collagen type I
SDs: Standard deviations
