# Supplementary material for: Graphene oxide-modified silk fibroin/nanohydroxyapatite scaffold loaded with urine-derived stem cells for immunomodulation and bone regeneration
Source: Stem Cell Res Ther. 2021 Dec 4;12:591. doi: 10.1186/s13287-021-02634-w (PMC8642892; doi:10.1186/s13287-021-02634-w)
Supplement: Supplementary file 1 — Additional file 1. Figure S1. TEM image of GO after dispersion. Scale bar = 0.5 μm. Figure S2. Histological evaluations for inflammatory response of scaffolds after 3 days of subcutaneous implantation, including H&E, CD68, iNOS, and CD206 staining. Scale bar = 200 μm. Figure S3. H&E, Masson, and Col I staining images of the specimens from each group at 6 weeks postsurgery. Scale bar = 200 μm. Figure S4. Images of CD68, iNOS, CD206, OCN, and CD31 staining of specimens after 6 weeks of implantation. Scale bar = 200 μm. Red arrows represent new vessels. Table S1. The percentages of O-C=O and C-N bond in scaffolds. [file 13287_2021_2634_MOESM1_ESM.docx]

**Graphene Oxide–Modified Silk Fibroin/Nanohydroxyapatite Scaffold Loaded With Urine-Derived Stem Cells for Immunomodulation and Bone Regeneration**

Jiachen Sun^[1, †]^, Lang Li^[2, †]^, Fei Xing^[1]^, Yun Yang^[1]^, Min Gong^[3]^, Guoming Liu^[4]^, Shuang Wu^[1]^, Rong Luo^[1]^, Xin Duan^[1]^, Ming Liu^[1]^, Min Zou* ^[5]^, and Zhou Xiang* ^[1]^

[1] *Dr. J. Sun, Dr. F. Xing, Dr. Y. Yang, Dr. S. Wu, Dr. R. Luo, Dr. X. Duan, Dr. M. Liu and Prof. Dr. Z. Xiang*
Department of Orthopedics, West China Hospital, Sichuan University, Chengdu, Sichuan 610041, P. R. China

[2] *Dr. L. Li*
Department of Orthopedics, Hospital of Chengdu Office of People’s Government of Tibetan Autonomous Region, Chengdu, Sichuan 610041, P. R. China

[3] *Dr. M. Gong*Department of Orthopedics, Hospital of Chengdu University of Traditional Chinese Medicine, Chengdu, Sichuan 610075, P. R. China

[4] *Dr. G. Liu*Department of Orthopedics, Affiliated Hospital of Qingdao University, Qingdao, Shangdong 266003, P. R. China

[5] *Dr. M. Zou*
Department of Orthopedics, Chengdu Second People’s Hospital, Chengdu, Sichuan 610017, P. R. China

[^†^] These two authors contributed equally to this work.

* Corresponding author:

*Prof. Dr. Z. Xiang,* Department of Orthopedics, West China Hospital, Sichuan University, Guoxue Lane 37, Chengdu 610041, Sichuan Province, P. R. China. Email: xiangzhou15@hotmail.com

*Dr. M. Zou,* Department of Orthopedics, Chengdu Second People’s Hospital, Chengdu, Sichuan 610017, P. R. China. Email: 286174326@qq.com





**Figure S1.** TEM image of GO after dispersion. Scale bar = 0.5 μm.


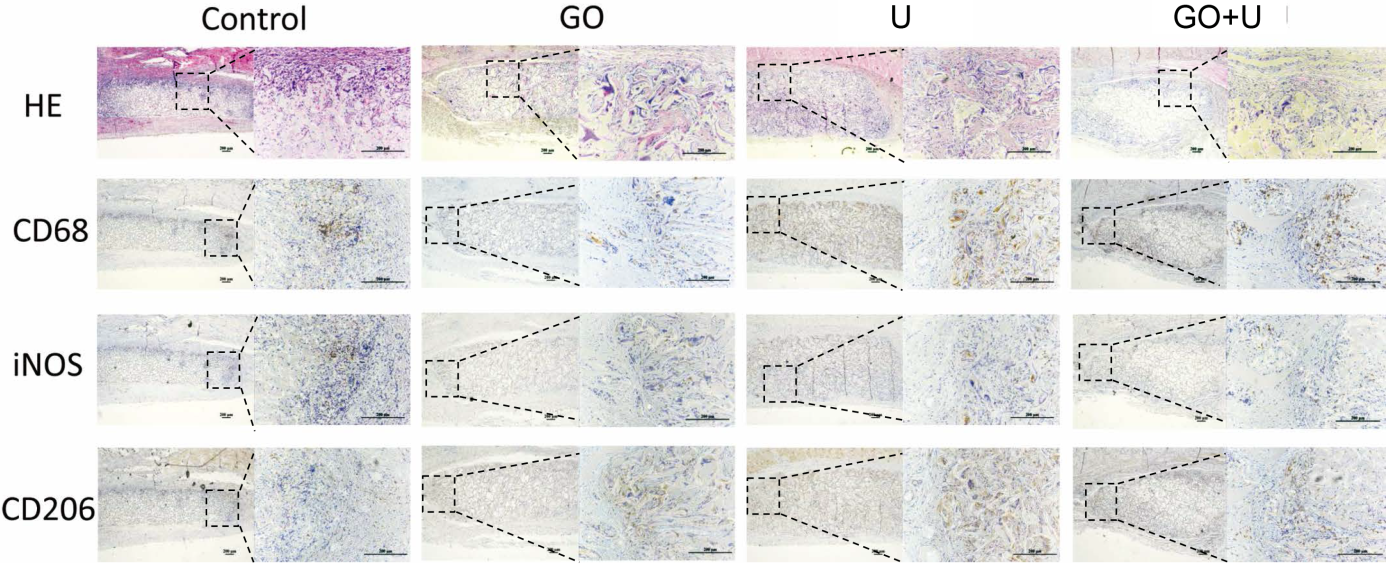


**Figure S2.** Histological evaluations for inflammatory response of scaffolds after 3 days of subcutaneous implantation, including H&E, CD68, iNOS, and CD206 staining. Scale bar = 200 μm.


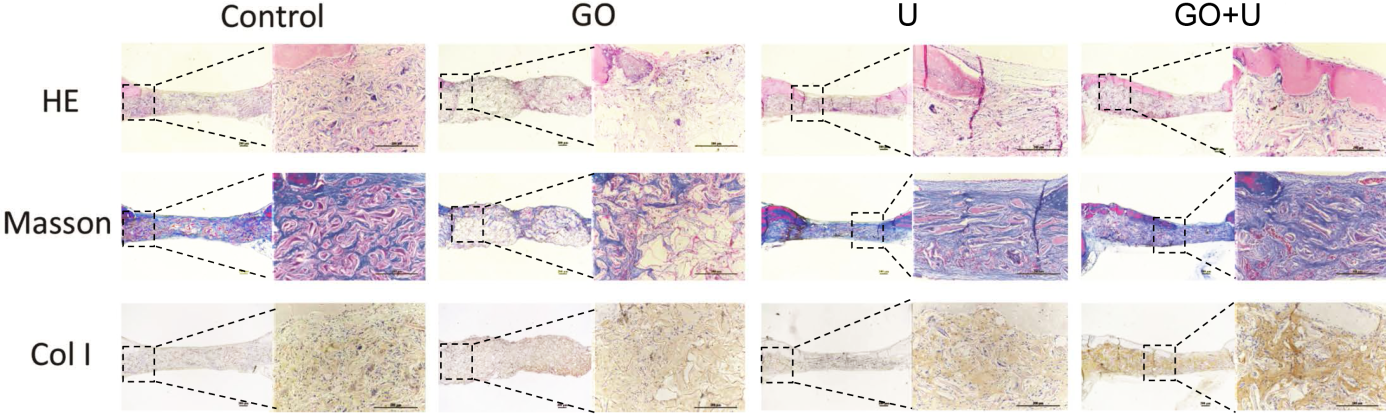


**Figure S3.** H&E, Masson, and Col I staining images of the specimens from each group at 6 weeks postsurgery. Scale bar = 200 μm.


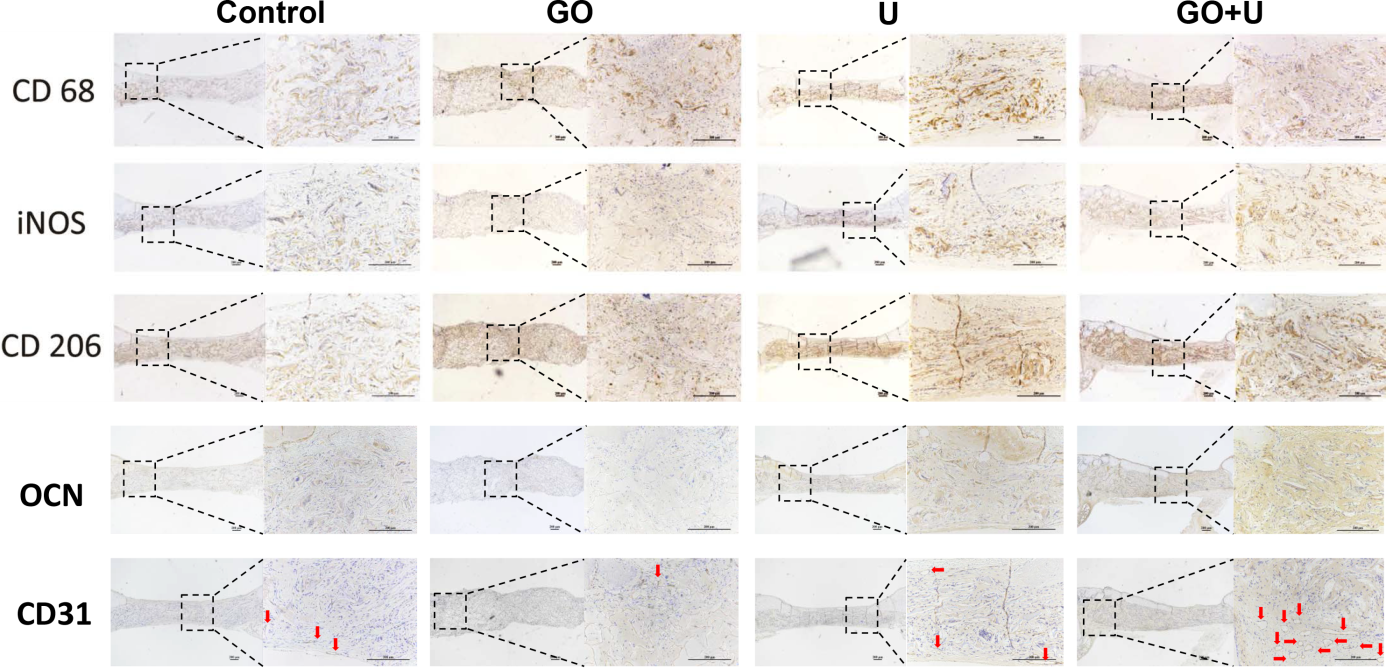


**Figure S4.** Images of CD68, iNOS, CD206, OCN, and CD31 staining of specimens after 6 weeks of implantation. Scale bar = 200 μm. Red arrows represent new vessels.

**Table S1.** The percentages of O-C=O and C-N bond in scaffolds

| Group | O-C=O (%) | C-N (%) |
| --- | --- | --- |
| Uncross-linked 0% | 6.50 ± 1.41 | 41.76 ± 0.09 |
| 0% | 1.65 ± 0.54 | 48.01 ± 4.35 |
| Uncross-linked 1% | 16.96 ± 1.53 | 40.64 ± 0.32 |
| 1% | 4.54 ± 0.88 | 42.47 ± 1.89 |

Scan spectra: O, 524–544 eV; C, 280–300 eV; N, 392–412 eV.
